# Making Physical Health a Priority: Introducing an In-House Perinatal Physical Health Clinic

**DOI:** 10.1192/bjo.2026.11411

**Published:** 2026-06-30

**Authors:** Hira Khurshid, Rebecca Harding

**Affiliations:** NAVIGO Health and Social Care CIC, Grimsby, United Kingdom

## Abstract

**Aims:**

Women accessing perinatal mental health services face marked physical health inequalities, exacerbated by pregnancy-related risks and the metabolic burden of psychotropic medication. Despite national guidance, compliance with physical health monitoring is poor, lifestyle interventions are inconsistently delivered, and engagement with external services is limited. This quality improvement project (QIP) aimed to address these gaps by establishing a dedicated in-house perinatal physical health clinic within our perinatal mental health service.

**Aims:**

To improve compliance with NICE recommended physical health monitoring for women prescribed antipsychotics; improve staff knowledge and confidence in physical health assessment and monitoring and increase access to holistic lifestyle and women’s health support.

**Methods:**

A baseline audit evaluated compliance with antipsychotic physical health monitoring (weight, blood pressure, glucose/Hbs-362c-glycated haemoglobin, lipid profile,ECG-electrocardiography, and movement disorder assessment) and the provision of lifestyle and women’s health advice. Staff knowledge and confidence were assessed using a pre-intervention survey. A twice-weekly physical health clinic was introduced, offering comprehensive physical health checks, structured lifestyle questionnaires and advice (diet, exercise, smoking and alcohol), and women’s health education (contraception and screening). Targeted teaching sessions were delivered to staff, and a repeat audit and post-intervention staff survey were completed following a four-week pilot.

**Results:**

Post-intervention audit data demonstrated substantial improvements in all monitoring domains, with completion rates for baseline physical health measures increasing from 40–60% to 100%. Monitoring for movement disorders and metabolic risk factors also significantly improved, whilst completion of lifestyle questionnaires increased from 0% to 100%, and documentation of dietary, contraceptive, and breast and cervical screening advice became universal. Staff confidence markedly improved, with only 14% initially reporting very good understanding of antipsychotic monitoring, compared to 100% reporting good to excellent understanding following teaching. All respondents reported increased confidence and would recommend the programme.

**Conclusion:**

Introducing a dedicated in-house perinatal physical health clinic resulted in significantly improved compliance with national guidance, enhanced staff confidence, and strengthened the provision of holistic care. This model demonstrates a sustainable, transferable and scalable approach to reducing physical health inequalities within perinatal mental health services.

